# Paul Dubois

**Published:** 1896-06

**Authors:** 


### PAUL DUBOIS.

We copy the following from a fly-leaf in the March number of *L'Odontologie*, Paris, giving the sad intelligence that this prominent worker in dentistry met a sudden death by an accident in April, the particulars of which are not given:

"P. Dubois, the distinguished editor of this journal, the devoted president of the General Association of Dentists of France, the learned professor of the Paris Dental School, died April 8, at Saint Germain en Laye, the victim of a terrible accident.

"The obsequies took place on Saturday, April 11, at Carrières Saint Dennis, in the midst of a large concourse of his colleagues and friends."

Dr. Dubois, as editor of *L'Odontologie* and as professor in the Dental College of Paris, earned an international reputation. The journal under his leadership secured a standing in the world of dental literature possessed by but few journals. While this will, doubtless, be continued with marked ability by the editorial staff, the readers will miss the energetic and able work of the editor-in-chief.

His professional labors in the furtherance of dental education have been continuous, and have, without doubt, been productive of the good results which follows all earnest efforts.

